# Detection of Epstein-Barr Virus Infection in Non-Small Cell Lung Cancer

**DOI:** 10.3390/cancers11060759

**Published:** 2019-05-31

**Authors:** Fayez Kheir, Mengmeng Zhao, Michael J. Strong, Yi Yu, Asuka Nanbo, Erik K. Flemington, Gilbert F. Morris, Krzysztof Reiss, Li Li, Zhen Lin

**Affiliations:** 1 Tulane University Health Sciences Center and Tulane Cancer Center, New Orleans, LA 70112, USA; fkheir@tulane.edu (F.K.); mzhao8@tulane.edu (M.Z.); yyu5@tulane.edu (Y.Y.); erik@tulane.edu (E.K.F.); gmorris2@tulane.edu (G.F.M.); 2 Department of Medicine, Tulane University Health Sciences Center, New Orleans, LA 70112, USA; 3 Department of Neurosurgery, University of Michigan, Ann Arbor, MI 48109, USA; mistrong@med.umich.edu; 4 Graduate School of Medicine, Hokkaido University, Sapporo, Hokkaido 060-8638, Japan; nanboa@med.hokudai.ac.jp; 5 Department of Medicine, Louisiana State University Health Sciences Center, New Orleans, LA 70112, USA; kreiss@lsuhsc.edu; 6 Institute of Translational Research, Ochsner Clinic Foundation, New Orleans, LA 70121, USA; lli@ochsner.org

**Keywords:** non-small cell lung cancer, NSCLC, Epstein-Barr virus, EBV, next-generation sequencing, NGS

## Abstract

Previous investigations proposed a link between the Epstein-Barr virus (EBV) and lung cancer (LC), but the results are highly controversial largely due to the insufficient sample size and the inherent limitation of the traditional viral screening methods such as PCR. Unlike PCR, current next-generation sequencing (NGS) utilizes an unbiased method for the global assessment of all exogenous agents within a cancer sample with high sensitivity and specificity. In our current study, we aim to resolve this long-standing controversy by utilizing our unbiased NGS-based informatics approaches in conjunction with traditional molecular methods to investigate the role of EBV in a total of 1127 LC. In situ hybridization analysis of 110 LC and 10 normal lung samples detected EBV transcripts in 3 LC samples. Comprehensive virome analyses of RNA sequencing (RNA-seq) data sets from 1017 LC and 110 paired adjacent normal lung specimens revealed EBV transcripts in three lung squamous cell carcinoma and one lung adenocarcinoma samples. In the sample with the highest EBV coverage, transcripts from the BamHI A region accounted for the majority of EBV reads. Expression of EBNA-1, LMP-1 and LMP-2 was observed. A number of viral circular RNA candidates were also detected. Thus, we for the first time revealed a type II latency-like viral transcriptome in the setting of LC in vivo. The high-level expression of viral BamHI A transcripts in LC suggests a functional role of these transcripts, likely as long non-coding RNA. Analyses of cellular gene expression and stained tissue sections indicated an increased immune cell infiltration in the sample expressing high levels of EBV transcripts compared to samples expressing low EBV transcripts. Increased level of immune checkpoint blockade factors was also detected in the sample with higher levels of EBV transcripts, indicating an induced immune tolerance. Lastly, inhibition of immune pathways and activation of oncogenic pathways were detected in the sample with high EBV transcripts compared to the EBV-low LC indicating the direct regulation of cancer pathways by EBV. Taken together, our data support the notion that EBV likely plays a pathological role in a subset of LC.

## 1. Introduction

Infectious agents have long been hypothesized to contribute to lung carcinogenesis [1,2]. The Epstein-Barr virus (EBV) is an extremely ubiquitous human virus and is causally associated with a variety of lymphoproliferative and neoplastic disorders, including nasopharyngeal carcinoma, Burkitt’s lymphoma, Hodgkin’s disease, and gastric cancer [3]. EBV may be associated with lung cancers (LC) since a higher EBV seropositivity was observed in LC patients compared to the one seen in the healthy control individuals [4,5]. EBV has been detected in the bronchoalveolar fluid collected from LC patients, indicating that the lung tissue may serve as a potential EBV reservoir [6]. The first EBV-positive LC case was reported by Begin and colleagues in 1987 [7] and histologically it seems that the EBV-positive LCs are more likely to be the primary pulmonary lymphoepithelioma-like carcinoma, a relatively rare form of non-small cell lung cancer (NSCLC) preferentially occurring in Asian patients [8,9,10,11,12,13]. Meanwhile, the presence of EBV in lung squamous-cell carcinomas (LUSC) and lung adenocarcinomas (LUAD) was also reported by several studies [14,15,16,17,18,19,20].

To date, the association of EBV and LC remains inconclusive, since quite a few studies of EBV in LC have produced negative results [21,22,23]. Notably, previous studies exclusively relied on traditional detection methods such as histology staining and polymerase chain reaction (PCR) to detect the EBV DNA and/or RNA. Although they are important methods, their intrinsic limitations (e.g., PCR false priming, usage of inappropriate/biased detection markers, etc.) can also lead to false discovery and/or controversy.

Recently, the next-generation sequencing (NGS) technology has been successfully applied to the discovery and interrogation of numerous cancer-associated pathogens. This approach utilizes an unbiased method to globally assess all the exogenous microbes within a cancer sample with high sensitivity and specificity. Several research groups including ours have successfully utilized NGS techniques and especially high-throughput RNA sequencing (RNA-seq) for the discovery and interrogation of exogenous pathogens associated with various types of cancers [24,25,26,27,28,29,30,31,32,33,34]. To date, this technology has helped us not only discover new tumor-associated pathogens, but also elucidate previous false discoveries.

Although EBV is likely associated with a subtype of non-small cell lung cancers, the conclusion remains controversial. To resolve this long-standing controversy, we utilized our unbiased NGS-based informatics approaches together with traditional molecular methods to investigate the role of EBV in a total of 1127 LC (including 1017 LC RNA-seq data sets plus 110 LC tissue samples). As far as we know, the magnitude of such screening work for EBV infection in LC has not been reported before, and we reasoned that the sample size should be sufficient for us to draw a definitive conclusion of whether EBV is associated with LC. We first analyzed the expression of EBV transcripts in LC cells by staining 110 LC plus 10 paired normal lung tissue sections. Strong EBV-encoded RNA (EBER) signals were detected in tumor cells in 3 LC samples. However, we did not detect any EBER signals in the tumor-infiltrating immune cells among 110 LC samples, indicating the scarcity of EBV-positive immune cells infiltrated in the LC tissues. Further, to investigate the presence of EBV in a broader lung cancer patient population, we utilized our sequencing-based approaches to examine the EBV infection in a total of 1017 human non-small lung cancer as well as 110 paired adjacent normal lung tissue samples from the NIH’s The Cancer Genome Atlas (TCGA) project. The presence of EBV was determined by its transcriptional activity using our recently created computational pipeline RNA CoMPASS [28,29,35]. EBV was detected in 4 out of 1017 NSCLC samples and the complete viral transcriptome structure was assessed. To the best of our knowledge, this is the first study to globally assess both EBV and its host transcriptomes in the lung cancer settings using RNA-seq. We for the first time revealed a type II latency-like viral transcriptome in the setting of LC in vivo. We also discovered high-level expression of viral BamHI A transcripts in LC, suggesting a functional role of these transcripts in LC development. In other EBV-associated cancers such as nasopharyngeal carcinomas, EBV is known to regulate the tumor immune microenvironment to facilitate tumor development. Interestingly, in the context of lung cancers, an increased immune cell infiltration was observed in the sample expressing high levels of EBV transcripts relative to samples expressing low EBV transcripts. Increased levels of immune checkpoint blockade factors were also detected in the sample with higher levels of EBV transcripts, indicating an induced immune tolerance. Lastly, our pathway analysis shows inhibition of immune pathways and activation of oncogenic pathways in the sample with high EBV transcripts, suggesting the direct regulation of tumor pathways by EBV.

Overall, our current study strongly indicates that EBV is not a major carcinogen for LC in general, but EBV may play a critical role to promote the development of a subset of lung squamous cell carcinoma and lung adenocarcinoma cases. Our data also led to significant insights into the EBV-host interactions and the mechanisms through which EBV promotes lung carcinogenesis. 

## 2. Results

### 2.1. EBV is Detected in Primary Non-Small Cell Lung Cancer Samples

**Figure 1:**
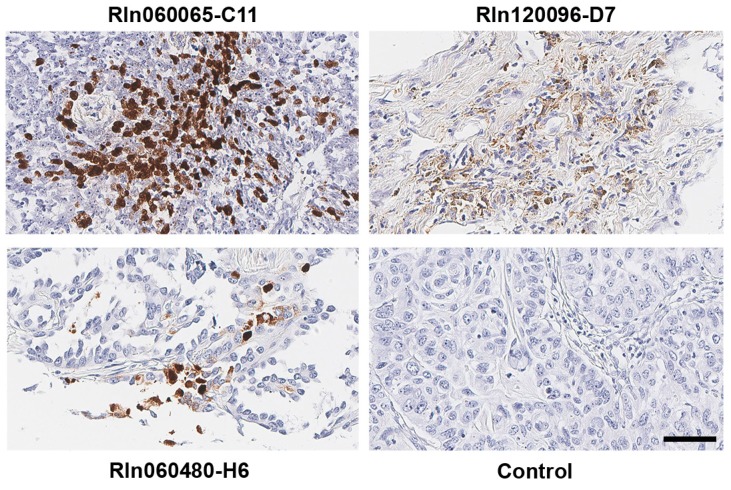
Detection of EBV marker small RNA EBERs in lung cancer cells. Images of paraffin-embedded human lung cancer probed for EBERs using in situ hybridization. EBERs (brown signal) were detected in three non-small cell lung cancer cases. Patient IDs were shown above or below each image. Control (patient ID# Rln060040-B11) represents an EBV-negative lung squamous cell carcinoma case. Scale bar: 50 µm.

To investigate EBV infection in lung cancers, we first analyzed 110 cases of lung cancer samples (including 40 cases of lung squamous cell carcinoma, 3 lung adenosquamous carcinoma, 48 lung adenocarcinoma, 4 bronchioloalveolar carcinoma, 3 large cell carcinoma, 8 small cell carcinoma and 4 malignant lung carcinoid tumors) as well as 10 paired normal lung samples. Expression of EBV marker small RNA EBERs [36,37,38] was measured by in situ hybridization. As shown in Figure 1, EBERs were detected in the non-small cell lung cancer cells, including one lung squamous cell carcinoma patient, one lung adenosquamous carcinoma patient, and one lung adenocarcinoma patient. However, we cannot detect EBV in other types of examined lung cancers such as small cell lung cancers or malignant lung carcinoid tumors or paired normal lung samples. Furthermore, we did not detect any EBER signal in the tumor-infiltrating immune cells, indicating the scarcity of EBV-positive immune cells in the tumor tissues. 

To further investigate EBV infection in non-small cell lung cancers, 1127 RNA-seq data sets from The Cancer Genome Atlas (TCGA) non-small cell lung cancer (NSCLC) project were downloaded from the NIH database, which carries the datasets from 501 primary lung squamous cell carcinoma (LUSC) samples, 516 primary lung adenocarcinoma (LUAD) samples, as well as 110 paired adjacent normal lung tissue samples. Because the analysis is highly computationally intensive, to improve the overall screening efficiency, virome analyses of all these polyA-selected RNA-seq datasets were performed by analyzing approximately 20 million of randomly selected reads (around 1/3 of the total reads which allow us to identify samples carrying a relatively high amount of EBV transcripts) from each sample using our automated RNA-seq exogenous organism analysis pipeline, RNA CoMPASS [28,29,35]. 

**Figure 2:**
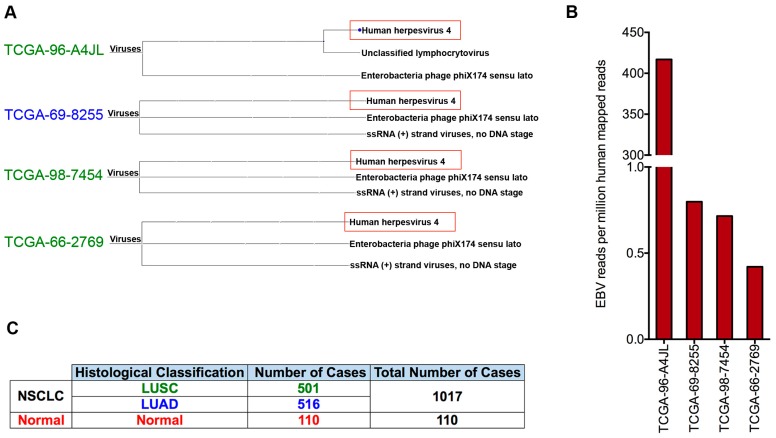
Detection of EBV in non-small cell lung cancer data sets. (**A**) Approximately 20 million of randomly selected RNA-seq reads from each of 1127 non-small cell lung cancer samples and tumor-adjacent normal lung tissue samples were analyzed using the RNA CoMPASS software. The virome branch of the taxonomy trees for the four EBV positive samples was generated using the metagenome analysis tool, MEGAN 4. (**B**) For more in-depth analyses of EBV reads, the entire sequence read file for each sample (~60–118 million reads) was aligned to the modified Akata-EBV genome and the hg38 human genome assembly using the STAR aligner. Of the EBV(+) samples, one sample (EBV-high) was identified as having high numbers of EBV reads, while three (EBV-low) were found to have low but detectable numbers of EBV reads. (**C**) Histology types of analyzed lung cancer specimens.

Most of the analyzed samples contained low levels of bacteriophage sequences (e.g., Enterobacteria phage phiX174), which are quality control spike-ins (Figure 2A). Among 1127 samples, EBV transcripts were detected in 4 non-small cell lung cancer datasets, but not detected in any paired control normal lung tissue samples (Figure 2A). Further, we did not detect any other known human pathogens in the EBV-positive LC samples. To thoroughly examine the EBV transcription in these EBV-positive datasets, the complete sequencing file for each EBV-positive tumor (~60–118 million reads) was aligned directly to the human reference genome (GRCH38/hg38) plus a modified Akata strain of the EBV genome that was split between the BBLF2/3 and the BGLF3.5 to accommodate coverage of splice junctions for the LMP-2 viral gene using the STAR aligner. As shown in Figure 2B (also see Table S1 in the supplementary material for the number of mapped viral and human reads), we found that sample TCGA-96-A4JL has the highest EBV read number (>400 reads per million human mapped reads), whereas the other 3 EBV-positive samples have relatively low EBV read numbers (around 1 read per million human mapped reads). Hereafter, we classified the sample TCGA-96-A4JG as EBV-high, and the other three EBV(+) NSCLC as EBV-low.

Given the rigorous diagnosis procedure of the TCGA, the chance of misdiagnosis of these NSCLC samples is slim. To further ensure the identity of these samples, here we set out to confirm the molecular origin of these EBV(+) NSCLCs. The cellular gene expression signatures of these four EBV(+) NSCLCs were analyzed by the unsupervised hierarchical cluster analysis together with six EBV(+) nasopharyngeal carcinoma (NPC) samples (our unpublished RNA-seq data). As shown in Figure S1 in the supplementary material, the four EBV(+) NSCLCs form their own branch which is well separated from the NPC branch. Thus, our data further confirm the diagnosis of the four EBV(+) NSCLCs and they are unlikely the metastasis from other EBV-associated epithelial tumors. 

Taken together, our virome screening work in a large number of LC indicates that EBV is not likely an etiological factor for the majority of the LC cases. However, the detection of EBV in a number of LC samples indicates that EBV may be associated with a subset of lung squamous cell carcinoma and lung adenocarcinoma cases.

### 2.2. Type I EBV is Detected in EBV(+) NSCLC

Based on the variation of the genomic sequence, geographic distribution, and virulence, there are two main types of EBV strains: Type I EBV and Type II EBV. Generally, the Type I EBV is more prevalent and also more virulent than the Type II EBV strain. We and others previously reported that among the EBV latency genes, EBNA-2, EBNA-3A, EBNA-3B, and EBNA-3C are uniquely and significantly less conserved between type I and type II EBV strains, but nevertheless show strong intra-strain conservation [26]. To identify the types of EBV that infected these viral associated lung cancers, we developed an in-house computational pipeline to genotype the EBV strains. More specifically, non-human reads from each EBV(+) samples were extracted and then aligned sequentially to the Type I Akata-EBV and Type II AG876-EBV reference genomes (NCBI genbank # DQ279927.1). Reads mapped to the unique EBNA-2/EBNA-3 regions of each EBV type were identified and the EBV type was determined by the number of viral reads mapped to these strain-determining regions. Since only the EBV-high sample has enough read coverage to allow an accurate genotyping call, we only analyzed the EBV-high sample and we found that the EBV-high NSCLC sample was Type I in accordance with the fact that all the analyzed lung cancer samples were collected in the Type I EBV endemic region (see Table S2 in the supplementary material). 

### 2.3. EBV Transcriptome Analysis

To the best of our knowledge, comprehensive analyses of the EBV transcriptome in the lung cancer setting have not been reported. We reasoned that the depth of coverage in these EBV(+) NSCLC RNA-seq data sets was sufficient for EBV transcriptome analysis. The EBV transcript quantification was then conducted by using the transcriptome analysis software, RSEM with a modified Akata-EBV genome. Notably, since the sequencing libraries were generated from polyA selected RNA, viral EBER transcripts were precluded during the library preparation. 

**Figure 3:**
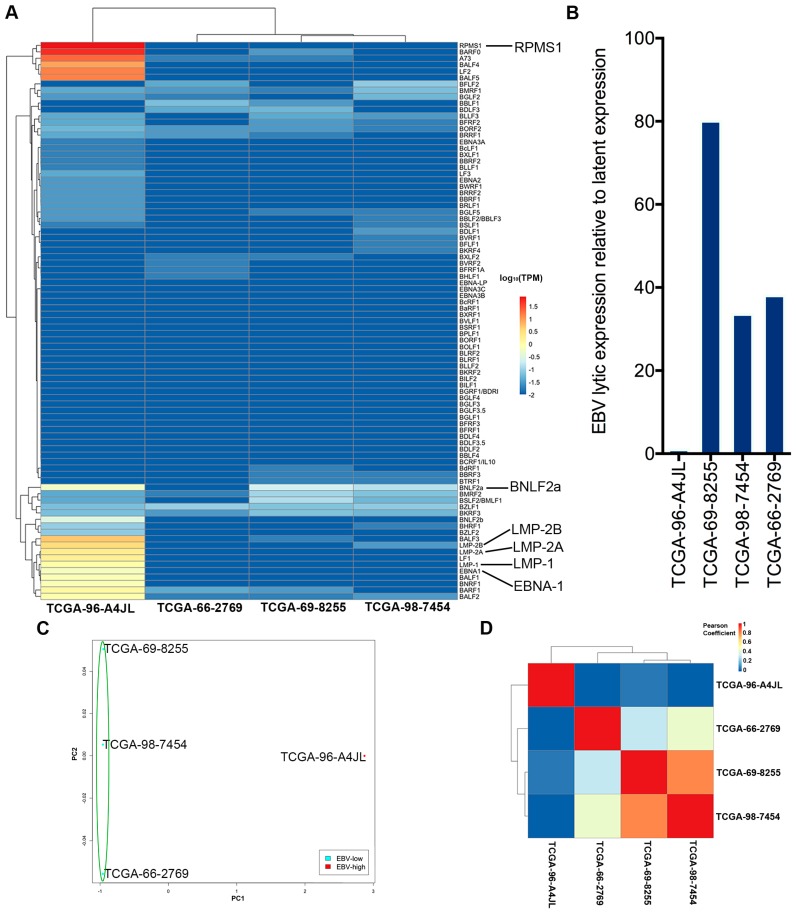
EBV gene expression analysis. (**A**) A heatmap shows EBV transcript levels for the four EBV(+) lung cancer samples. Unsupervised hierarchical clustering separated the EBV-low and EBV-high samples. (**B**) The ratio of EBV lytic-to-latent gene expression for each EBV(+) sample. (**C**) Principal component analysis (PCA) of variability among EBV(+) NSCLC samples based on EBV gene expression. Each point represents one sample, with color indicating sample groups as described in the figure legends. (**D**) A plot of the EBV gene expression pattern determined by correlation analyses of the EBV(+) samples.

Our results showed that the EBV-high sample has a distinct viral gene expression pattern compared to the EBV-low samples (Figure 3A). In the EBV-high sample, genes from the BamHI A region expressed at high levels, including RPMS1, A73, etc., but these genes were barely detectable in the EBV-low samples. Furthermore, other key EBV latent gene transcripts including EBNA-1, LMP-1, LMP-2A, and LMP-2B were also detected in the EBV-high sample, but not in the EBV-low samples. Meanwhile, EBV type III latency genes, EBNA-3A, -3B, -3C and -LP were not detected in any of these samples. Thus, our data indicate that the EBV-high sample exhibits a Type II-latency-like infection in the lung cancer setting. 

EBV immediate-early lytic transactivator BZLF1 is similarly detected in all four EBV-positive samples (Figure 3A). Despite the detection of BZLF1 reads in the EBV-high sample, a remarkable absence of reads for most other downstream lytic genes was observed in this sample. Consistently, as shown in Figure 3B, we plotted the ratio of lytic gene transcripts to the latent gene transcripts and found that the EBV-low samples show high lytic-to-latent ratio. The lack of distinct latency-gene expression together with the observed overall low EBV transcript levels for the samples with low EBV read numbers raise the possibility that the finding of EBV in these EBV-low lung cancer samples is less consequential than it is in the EBV-high sample. Further, we cannot rule out the possibility that the low level EBV reads were partially derived from the low-level reactivation in infiltrating latent B-cells, although our EBER staining did not show any evidence of EBV-positive immune cells infiltrating in the LC tissues. 

**Figure 4:**
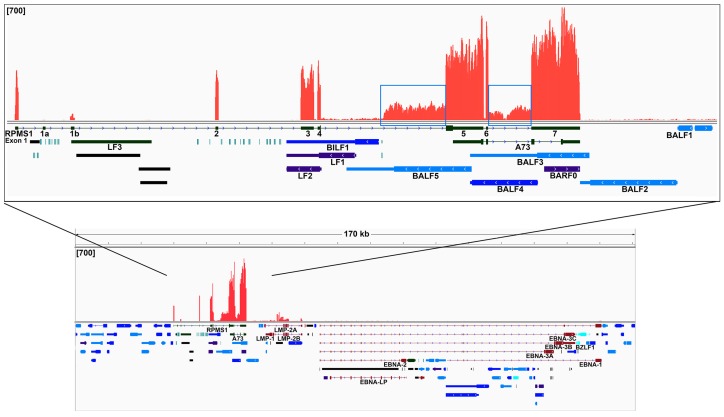
The EBV transcriptome in lung cancer. EBV genome coverage data for the EBV-high NSCLC is shown using the Integrative Genomics Viewer (IGV) based on the modified Akata-EBV genome. The modified EBV Akata genome was split between the BBLF2/3 and the BGLF3.5 lytic genes rather than at the terminal repeats to accommodate coverage of splice junctions for the latency membrane protein LMP-2. The *y*-axis represents the number of reads at each nucleotide position. The scale for the sample is set to a maximum read level of 700 reads. Blue features represent lytic genes, red features represent latent genes, green features represent potential noncoding genes, aquamarine features represent microRNAs, and black features represent non-gene features (e.g., repeat regions). Inset: Detailed read coverage data for the RPMS1/BamHI A region of the EBV genome.

To further elucidate global differences in EBV gene expression patterns in these EBV(+) lung cancers, we performed an unsupervised hierarchical cluster analysis, a correlation analysis, and a principal component analysis of the viral gene expression data. Consistently, the samples with a low number of EBV read counts were found to group together (Figure 3C,D). In addition, visualization of reads across the EBV genome using the Integrative Genomics Viewer (IGV) software showed latency-gene peaks in the sample with high EBV read counts (Figure 4). In contrast, only scattered reads were observed across the entire genome in the samples with low EBV read counts (see Figure S2 in the supplementary material). 

### 2.4. Analysis of the Highly Transcribed BamHI A Region

Our data show that the BamHI A region is the most actively polyA transcribed region of the EBV genome (Figure 4). As shown in the coverage data, we found that most of the RPMS1 and A73 gene exons show excellent read coverage. Meanwhile, additional coverage was also observed for the regions carrying the leftward transcribed genes such as BALF3, BALF4, BALF5, BILF1, LF1, and LF2. Coverage across these leftward genes is unexpected since they are classified as lytic genes and are not expressed during the latency. Because all the sequencing data sets were not developed from strand-specific libraries, we cannot differentiate the leftward transcripts from the rightward transcripts. But since a similar phenomenon has also been discovered in EBV(+) gastric cancer samples using strand-specific RNA-seq data [28], we reasoned that the transcription observed across this region in the lung cancer specimen is likely rightward oriented and largely related to the RPMS1 and/or A73 but not BALF3, BALF4, BALF5, BILF1, LF1, or LF2. 

As shown in the boxed regions in Figure 4 inset, we observed some significant coverage across the introns between exons 4 and 5 as well as exons 6 and 7 of the RPMS1 gene. We reasoned that the coverage is unlikely due to the spliced-out intron fragments for two reasons: (i) The sequencing library was generated from polyA selected RNA; (ii) we did not detect any coverage of the first 6 RPMS1 introns. Thus, the coverage between exons 4 and 5 as well as between exons 6 and 7 may reflect novel and uncharacterized exons or transcripts. The coverage between exons 6 and 7 may be derived from a novel intron-bearing RPMS1 isoform. Meanwhile, since the coverage between exons 4 and 5 largely initiates at the middle of this intron, it suggests that this is likely a transcription initiation site or a splice acceptor site. Further, since our subsequent splicing analysis did not detect any candidate splicing events at the beginning of this intron coverage, we reasoned that this read coverage may result from a transcription initiation from an unknown upstream promoter.

**Figure 5:**
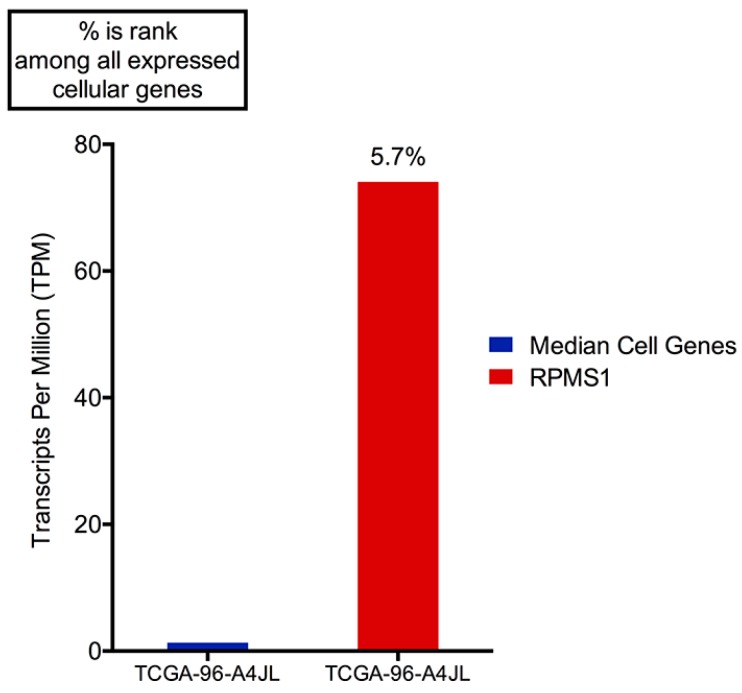
EBV transcripts from RPMS1 are among the highest expressed genes in EBV(+) NSCLC. Transcripts per million (TPM) values calculated using reads across all RPMS1 exons are shown with respect to the median expression of all expressed cellular genes (expressed genes defined as cellular genes with greater than 0.01 TPM). The percentage values above the RPMS1 bar represents the rank of RPMS1 expression among all expressed cellular genes.

In the EBV-high lung cancer sample, greater than 96.3% of all EBV reads align to the BamHI A region. Furthermore, RPMS1 exon coverage ranks within the top six percent of the expressed cellular genes in the EBV-high lung cancer sample and is forty-five times more than the median cellular gene expression level (Figure 5). Thus, we concluded that the expression of this region is not only high compared to other EBV-encoded genes, but the expression is also high relative to cellular genes. However, within the BamHI A locus, we did not detect LF3 gene expression in the in vivo lung cancer dataset, although we previously reported high levels of LF3 in Burkitt’s lymphoma [24]. 

**Figure 6:**
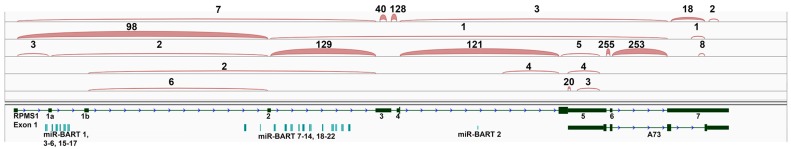
Alternative splicing in the EBV BamH1 A region in EBV-high NSCLC. RNA-seq data of the EBV-high NSCLC were analyzed using the STAR aligner and were aligned to the modified Akata-EBV genome to obtain splice junction information. Junctions were visualized using the Integrative Genomic Viewer (IGV). The thickness of the red junction features correlates with the number of reads for the respective junction. The number of junction spanning reads for each junction is indicated above each junction feature.

We next analyzed the splicing events in the BamHI A region, and the alignment was conducted using the STAR aligner. As shown in Figure 6, we found that the most abundant splice junction reads reflect the predominance of splicing from exons 1-2-3-4-5-6-7. However, significant splicing events were detected within exons 3, 5, and 7. Further, exons 1a, 1b, and 2 were also used to generate alterative splicing, although to a lesser degree. Notably, we also detected some splicing events (coverage = 4) that initiate from the middle of the newly identified coverage in the intron between exons 4 and 5 to the start of exon 5, suggesting that some transcripts splice to exon 5 whereas some read through to exon 5.

### 2.5. Analysis of the Viral circRNAs

Using both computational and wet lab techniques, we have recently discovered and validated a repertoire of EBV encoded circular RNAs (circRNA) in EBV infected cell lines and clinical samples [39]. Here, we’d like to investigate if any backsplicing events of viral transcripts occurred in EBV(+) lung cancers. The RNA-seq data set of the EBV-high NSCLC was then analyzed with the find_circ pipeline and a total of 4814 unique backsplice junctions were identified and 3 of them belong to the EBV. Interestingly, all these EBV circRNA candidates were derived from the RPMS1 region (Table 1). Notably, since the RNA-seq library was constructed to enrich poly-adenylated RNA but not the circRNA, we reasoned that the true abundance of viral circRNA in EBV(+) LC should be much higher than what we observed here.

### 2.6. Differential Cellular Gene Expression Profiles in EBV-High and EBV-Low Lung Squamous Cell Carcinoma

We hypothesized that EBV may promote lung cancer development by altering certain oncogenic pathways. Nevertheless, the mechanism that EBV manipulates these oncogenic pathways may be different from the pathway disruption mechanism utilized in the absence of EBV such as through gene/chromosomal mutations. Because cellular gene expression usually responds to the altered signaling mechanisms, we thus can use differences in gene expression patterns to classify cell populations as well as reveal signaling events within certain cell populations. To elucidate the influences of EBV-dependent alterations in oncogenic pathways, we first assessed global cellular gene expression in all 4 EBV(+) lung cancer samples. We did not include EBV gene expression data in this analysis to ensure that clustering occurred based solely on differences in cellular gene expression and will not be affected by the biases incurred by the signatures of EBV gene expression. 

**Figure 7:**
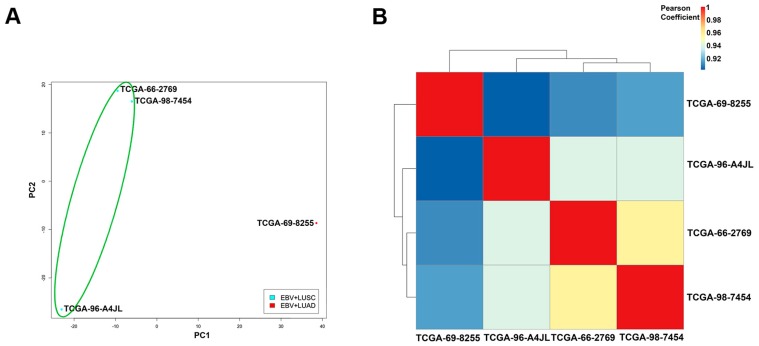
Dimension reduction, correlation and cluster analysis of cellular gene expression data of the EBV(+) NSCLC. (**A**) Principal component analysis (PCA) of variability among EBV(+) NSCLC samples based on cellular gene expression. Each point represents one sample, with color indicating sample groups as described in the figure legend. (**B**) Correlation analysis of the EBV(+) NSCLC samples with the plot showing the correlation to the cellular gene expression pattern.

Notably, when these samples were analyzed using principal component analysis (PCA), the three EBV(+) lung squamous cell carcinoma (LUSC) samples displayed slight differences (based on the distance along the PC1 axis) (Figure 7A), while the EBV(+) lung adenocarcinoma (LUAD) sample showed significant differences and large separation from the LUSC group. In accordance, our correlation and cluster analysis showed that EBV(+) LUSC samples form a well-separated branch, and the EBV(+) LUAD sample forms another main branch (Figure 7B). These data indicate that LUAD and LUSC are distinct molecular subtypes of NSCLC and carry their own unique molecular signatures. Thus, since only the LUSC samples carry both high levels and low levels of EBV in the analyzed NSCLC data sets, to better determine the role of EBV infection in the development of NSCLC, we restricted our analysis to the EBV-high and EBV-low LUSC samples. Strikingly, as shown in the correlation and cluster plot (Figure 7B), within the EBV(+) LUSC groups, the two EBV-low LUSC samples clustered together and were well-separated from the EBV-high LUSC sample. Thus, a different gene expression pattern occurred in the EBV-high sample.

The count data of human transcripts from the LUSC with high levels of EBV reads were subsequently compared to the LUSCs with low levels of EBV reads using the EB-Seq algorithm [40]. Notably, the empirical Bayes hierarchical algorithm of EB-Seq allows us to calculate statistically significant gene alterations between 2 groups. Importantly, it allows a minimum of one sample in each group and the gene alteration will be determined as statistically significant if the false discovery rate (FDR) is less than 0.05. We found that a total of 4994 cellular genes show statistically significant differential expression (FDR < 0.05) in the EBV-high LUSC sample relative to the EBV-low LUSC samples. 

We next used the Ingenuity Pathway Analysis (IPA) software to analyze pathways and known molecular functions associated with differentially expressed cellular genes. In the EBV-high LUSC sample, the most activated canonical pathway was the BRCA1-mediated DNA damage response (Table 2). It suggests that EBV infection in LUSC cells may promote BRCA1 signaling. This observation is consistent with previous studies reporting that BRCA1 is involved in the innate sensing of herpes virus DNA and also involved in EBV replication [41,42]. In addition, TNF-receptor signaling was also activated in the EBV-high LUSC cells, which may be due to the expression of viral LMP-1, a constitutively activated truncated form of the TNF receptor. CDK5 signaling can be activated by EBV infection and viral EBNA-2 expression in lung cancer via upregulation of the CDK5 activator p35 [43]. Further, multiple cancer associated signaling pathways were activated in the EBV-high LUSC, including G2/M and G1/S cell cycle control, the p53, HIPPO, and Sirtuin signaling pathways (Table 2). 

More than half of the top inhibited canonical pathways in the EBV-high LUSC were involved in immune regulation (Table 3). EBV infection may repress leukocyte extravasation and inhibit neutrophil signaling, B cell activation, as well as signaling mediated by multiple key cytokines such as TGF-beta and IL-8. 

The IPA analyses allowed us to identify the candidate master upstream regulators (UR) which were likely responsible for the observed alterations in cellular signaling. Interestingly, four of the fifteen activated URs are oncogenic miRNAs (Table 4). We previously showed that EBV latent infection induces microRNA miR-155 expression and activities in various EBV model cell systems [44,45] and the activation of miR-155 in EBV-high LUSC may be caused by similar mechanisms. Notably, all of the identified activated URs are either oncogenes or associated with tumor development (Table 4). 

The majority of the inhibited URs are cytokines or molecules involved in the immune response (Table 5), which is consistent with the findings that many immune pathways were also inhibited in the EBV-high LUSC. For example, the top inhibited molecule, TGFB3, may correlate with down-regulation of TGF-beta target genes by EBV EBNA-1 protein. This process is known to promote progression of Hodgkin’s lymphoma [46]. Thus, the inhibition of both immune pathways and immune URs may help induce tolerance to infiltrating immune cells in EBV-high LUSC. 

### 2.7. Elucidation of the Tumor Immune Microenvironment

Recent studies have used computational deconvolution approaches to elucidate the relative immune cell content in the tumor microenvironment from gene expression data. One such method, known as CIBERSORT, has shown consistent agreement between immune cell classification by deconvolution of RNA-seq data and flow cytometry [47]. The CIBERSORT method assumes that the gene expression pattern of an unknown bulk biospecimen can be interpreted by the weighted sum of the cell-type specific patterns that it comprises. An accurate deconvolution is largely dependent on the signature gene expression panel (SGEP) utilized. The expression levels of genes in the panel should be quantified well enough to distinguish between the types of cells contained in the mixed population. The default SGEP of the CIBERSORT software is based on the microarray data, which is not fully compatible with the RNA-seq data derived from lung tumors. Therefore, we developed our own RNA-seq based SGEP (namely LM18) which is derived from RNA-seq data of 18 immune cell types. 

**Figure 8:**
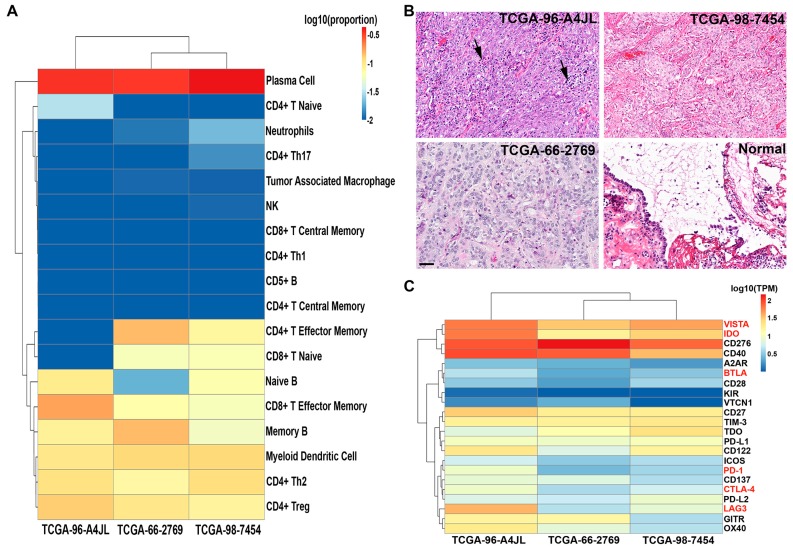
Immune infiltration status of EBV(+) NSCLCs. (**A**) Fractions of immune cell subsets in EBV(+) NSCLC samples inferred from gene-expression data using CIBERSORT. CIBERSORT empirical *p* value, *p* < 0.001. (**B**) Representative images of hematoxylin and eosin staining of EBV(+) NSCLC and adjacent normal lung samples. Arrowheads point to the infiltrating immune cells. Scale bar: 50 µm. (**C**) A high EBV level is associated with enhanced expression of immune checkpoint molecules in EBV(+) NSCLC samples. Heatmap shows transcripts levels of known cellular checkpoint molecules in EBV(+) NSCLC samples. Checkpoint molecules that were significantly up-regulated in the EBV-high sample are highlighted in red. Unsupervised hierarchical cluster analysis shows the separation of EBV-low and EBV-high samples.

To dissect infiltration of specific immune cell subsets, we then set out to deconvolve the gene expression data of EBV(+) LUSCs using CIBERSORT. The largest immune cell components corresponded to B cells, CD4+ and CD8+ T cells (Figure 8A). The unsupervised hierarchical cluster analyses showed that the EBV-low LUSCs had similar immune cell components, which differed from the EBV-high LUSC. The EBV-high LUSC had a higher proportion of CD8+ T effector and CD4+ T naïve cells as well as a lower proportion of CD8+ T naïve and CD4+ T effector cells relative to the EBV-low LUSCs. 

H&E-stained tissue sections (Figure 8B and see Figure S4 in the supplementary material for a high-resolution image) revealed that the EBV-high LUSC had a higher level of infiltrating immune cells compared to the EBV-low LUSCs. Although more observations will be necessary to confirm this correlation, these data are consistent with the concept that higher levels of EBV infection may promote infiltration of immune cells into the lung tumors. 

To further investigate the potential role of EBV in the host immune regulation, we analyzed the expression levels of known immune checkpoint molecules, which regulate the activities of infiltrating immune cells and are involved in the tumor immune tolerance. Interestingly, our clustering analyses revealed that the EBV-low LUSCs have similar expression patterns of immune checkpoint molecules (Figure 8C). Relative to the EBV-low LUSCs, the EBV-high LUSC expressed elevated levels of key inhibitory checkpoint molecules such as IDO, PD-1, CTLA-4, LAG3, BTLA, and VISTA. This finding agrees with the hypothesis that EBV may promote the expression of these checkpoint molecules and thereby promote tumor escape from the immune surveillance. 

The total amount of immune checkpoint molecules is determined by their expression within tumor cells and infiltrating immune cells. Recent studies have shown that tumor cell-intrinsic checkpoint molecules such as IDO, PD-1, CTLA-4 and VISTA likely play important roles in the development of non-small cell lung cancer [48,49,50,51]. Next, we set out to elucidate if EBV can directly induce these checkpoint molecules expression in lung cancer epithelial cells. To the best of our knowledge, a lung cancer cell line carrying naturally infected EBV has not been reported. To discover any existing EBV(+) lung cancer cell lines, we then screened EBV infection using RNA-seq data of 184 known lung cancer cell lines from the CCLE (Cancer Cell Line Encyclopedia) cohort (for the list of analyzed cell lines, see Table S3 in the supplementary material). The total sequencing reads of each dataset were aligned to a reference genome containing a human genome (hg38; Genome Reference Consortium GRCH38) plus a modified Akata-EBV genome using the STAR aligner. The results show that no EBV transcriptional activity was detected in these datasets. Since there’s no available EBV(+) lung cancer cell line, we then decided to introduce recombinant EBV genomes into the lung cancer cells. A lung squamous cell carcinoma cell line, NCI-H1703 was then transfected with plasmids carrying either recombinant EBV M81 strain (rM81) or B95.8 strain (rB95.8), respectively. The EBV(+) cells can be monitored based on their constitutive expression of green fluorescence protein (GFP) carried by the recombinant EBV (rEBV) genomes. Forty-eight hours post-transfection, cells were harvested and around 10% of cells were positive for EBV infection based on the GFP signal (Supplementary Figure S5A). Total RNAs were then extracted for the qRT-PCR analysis. Compared to the control group, high levels of EBV EBER transcripts were detected in rEBV-transfected cells, indicting a high EBV transcriptional activity (Supplementary Figure S5C). As shown in Figure S5C, EBV RPMS1 was also detected in EBV(+) cells, which is consistent with our RNA-seq data as shown in Figure 3. Compared to the EBV negative NCI-H1703 control cells, cellular checkpoint molecules such as IDO, PD-1, CTLA-4, and VISTA were strongly induced in rB95.8 EBV(+) lung cancer cells (Supplementary Figure S5B). Meanwhile, PD-1 and CTLA-4 were also induced in rM81 EBV(+) lung cancer cells. The lower induction of cellular checkpoint molecules by rM81 EBV may be due to virus’ high lytic activity, as evidenced by the high levels of lytic inducer BZLF1 in rM81 EBV(+) cells (Supplementary Figure S5C). Meanwhile, neither EBV strain can effectively induce LAG3 and BTLA expression in lung cancer cells, suggesting that these molecules were mainly expressed within the infiltrating immune cells in the tumor tissue. Thus, our results further support the notion that EBV infection may manipulate the cellular checkpoint molecule expression in lung cancer cells, and subsequently contribute to lung carcinogenesis.

## 3. Discussion

Although smoking is a key risk factor for lung cancer development, the incidence of lung cancer is slowly declining even after the dramatic reduction of smoking through public health awareness movement. Only 10–20% of total smokers develop lung cancer [52]. Further, around 15% of male lung cancer patients and 53% of female lung cancer patients are never smokers, and lung cancer is believed to be the 7th most common cause of cancer death in never smokers [53], indicating other etiological factors for lung cancer development.

EBV has been previously proposed to be associated with certain subtypes of lung cancer, but that conclusion was exclusively based on the results from traditional viral screening methods such as PCR. Due to the inherent limitations of those traditional screening methods (such as PCR priming issues, usage of inappropriate/biased detection markers, etc.), the reported EBV-lung cancer association was questionable. Here, in addition to the PCR-based method, we utilized an RNA-seq based informatics approach to comprehensively interrogate the involvement of EBV in the lung cancer in an unbiased and more accurate manner. Our analyzed data sets were derived from samples collected in nine countries such as the United States, Germany, Australia, etc. The patient population is not restricted to a particular race but includes Caucasians, Blacks, and Asians. The total number of RNA-seq data sets is 1127 (Figure 2C). To the best of our knowledge, the magnitude of such screening work for EBV infection in lung cancer has not been reported before. 

By doing in situ hybridization, we detected EBERs in non-small lung cancer cells but not the infiltrating immune cells. It suggests that EBV can indeed infect lung cancer cells. Meanwhile, our virome screening analyses of the TCGA data sets demonstrate that 4 cases with EBV positivity, but only the EBV-high sample undoubtedly represents a *bona fide* latent EBV infection of tumor cells. Although less likely, we cannot totally rule out the possibility that EBV reads detected in the 3 EBV-low samples are partially derived from the infiltrating EBV-positive immune B cells. If that is the case, the true EBV infection rate in the analyzed TCGA cohort is no more than 0.4%. This low EBV infection rate indicates that EBV is unlikely to play a significant role in the development of lung cancers in general, but may contribute to the development of a subset of lung cancer cases. In areas where EBV-associated cancers are endemic, such as sub-Saharan Africa and Southeast Asia, the connection between EBV and lung cancer may be more prevalent.

Another rational explanation for the observed low EBV incidence rate is that EBV may utilize the hit-and-run strategy to infect lung epithelial cells, which subsequently contributes to lung cancer development [54]. The transient presence of EBV genomes can potentially cause some genetic scars in the host cells and lead to a permanent alternation of cellular gene expression and promote tumorigenesis. In accordance, a recent study reported that EBV may promote breast cancer development using the hit-and-run mechanism [55]. 

Notably, the only EBV-high sample from the TCGA cohort was collected from an Asian female patient (Table 6). This is consistent with the notion that the Asian population is more susceptible to EBV-associated lung cancer [20,56]. Our results support previous findings that in the lung cancer setting EBV is not restricted to the lymphoepithelioma-like carcinoma (LELC) subtype [14,15,16,17,18,19,20]. Moreover, the EBV-high sample was collected from a never-smoking patient, indicating that EBV may promote lung carcinogenesis in a smoking independent manner. Although speculative, these findings offer a plausible explanation for the high incidence of lung cancer in never smoking Asian women [57].

Since EBV may underlie the pathogenesis of some lung cancers, it is important to determine how EBV interacts with the host cells. Our analyses detect a type II latency-like EBV transcriptome in lung cancer, which mimics the viral gene expression pattern seen in the EBV associated gastric cancer. The high levels of BamHI A transcripts detected in the EBV-high lung cancer sample are consistent with true EBV latency, since BamHI A transcripts are more highly expressed in the infected epithelial cells than in B cells. We also detected transcripts from two novel regions within the BamHI A segment, the region between exons 4 and 5, as well as between exons 6 and 7. Since the coverage of reads starts in the middle of the intron between exons 4 and 5, there is likely a new transcription initiation site or a new splice acceptor site within this intron. We did not detect any splicing event near the beginning of this intron coverage. Therefore, we reasoned that a hidden upstream promoter may initiate this transcript and it is responsible for the observed coverage. Further analysis is warranted to characterize this region for new genes or new transcript isoforms. 

Our analyses of RNA-seq evaluate transcript structures and quantify the expression of BamHI A region genes compared to other viral and cellular genes. Although previous studies have been unable to detect protein from naturally expressed BamHI A rightward transcripts [58,59], the high expression level of these transcripts in EBV-high NSCLC sample suggests a functional role in lung cancers, possibly as long non-coding RNAs (lncRNA), which has been previously proposed in the EBV(+) gastric cancers [60]. Many lncRNAs function in complexes that repress transcription, which raises the possibility that the rightward BamHI A transcripts function as lncRNAs that selectively repress cellular gene expression in EBV-high NSCLCs. These rightward BamHI A transcripts also encode at least 44 intronic BART microRNAs (miRNAs). The function of these BART miRNAs in the EBV’s life cycle and in EBV-associated cancers have been previously explored [61]. The high expression level of the BamHI A rightward transcripts in lung cancer would facilitate a significant role in modulating the cellular phenotype by BART miRNAs in this tumor type. In addition, the new coverage region detected in the RPMS1 and A73 region indicates that additional rightward exons/genes are present within this region and they may similarly play a role in noncoding RNA-mediated modulation of cellular function. 

Previously, we and others have identified novel alternative splicing events of LMP2A in EBV associated cancers [62,63,64]. Here, the sequencing depth of the EBV-high LC allows us to further characterize the transcript structure of LMP2A in the setting of LCs. In accord with the previous observation in the EBV(+) ENKTL (extra-nodal NK/T-cell lymphoma) and CAEBV (chronic active EBV) samples, classical splicing event between the first exon (exon 1A) and exon 2 of LMP2A was not detected (Supplementary Figure S3). The read coverage of the intronic region next to the 5′ end of exon 2 indicates that an alternative promoter may be utilized to initiate the LMP2A transcription in EBV(+) lung cancers (Supplementary Figure S3 inset). Furthermore, a novel splicing event between splicing sites located within LMP2A exon 2 and RPMS1 exon 7 was also detected. Thus, together with previous findings, our data indicate that the alternative splicing of LMP2A may be more common than we previously expected and it may play important regulatory and functional roles in EBV’s life cycle and pathogenesis.

We detected a high level of EBV BNLF2a gene expression in the absence of significant expression of other viral lytic genes in the EBV-high NSCLC sample. BNLF2a is an early lytic phase protein that suppresses immune detection of the EBV infected cells by blocking antigen presentation through inhibition of peptide loading onto the major histocompatibility complex (MHC) class I molecules [30,65,66,67,68,69]. Previously, we found that BNLF2a is expressed in a good portion of EBV- associated gastric cancers and EBV(+) gastric cancer cell lines [28,30]. Expression of BNLF2a with EBNA-1 and LMP-2 in the absence of reactivation suggests a new latency program. Thus, a subset of lung and gastric cancers may possess an EBV-associated etiology characterized by immune tolerance promoted by BNLF2a. 

We reported previously that gastric carcinomas with high levels of EBV reads exhibit activation of distinctive pathways, compared to samples with low/no EBV reads [28]. Here, using this established approach in the EBV-high NSCLC sample, we detected activation of EBV-associated oncogenic pathways and inhibition of multiple tumor suppressors. Moreover, our computational assessment of immune cell infiltration was confirmed in the stained tissue section from the EBV-high sample. Despite this heightened influx of immune cells, EBV-positive tumor cells persist in the patient. We reasoned that the tumor may have successfully employed certain immune evasion strategies perhaps facilitated by BNLF2a that allow virus/tumor survival. First, the limited expression of viral protein-coding genes in the EBV-high sample likely contributes to the avoidance of viral antigen targeting [70]. Second, even though the EBV-encoded protein, EBNA-1 is required for viral episomal maintenance/replication and thereby must be expressed in proliferating cells, it encodes a glycine-alanine repeat domain to block its proteasomal processing for cytotoxic T-lymphocyte presentation [71,72]. Third, here we found elevated expression of multiple immune checkpoint inhibitors, such as IDO, PD-1, CTLA-4, LAG3, BTLA, and VISTA in the EBV-high NSCLC sample. These immune inhibitors may contribute to EBV-associated tumor immune tolerance. For example, IDO (indoleamine 2,4-dioxygenase), one of the top EBV-induced immune inhibitors, may inhibit the activities of cytotoxic T lymphocytes and NK cells by causing local tryptophan depletion in the tumor niche and thus help promote tumor survival [28,73,74,75], despite the enhanced immune infiltration in the EBV-high NSCLC. The recent development of antagonists for these immune checkpoint inhibitors in the tumor immunotherapy field may eventually help improve the prognosis of the EBV(+) NSCLC in the near future. 

Together, our current data support the notion that EBV likely plays a pathological role in a subset of NSCLC. However, due to the limitation of our study (especially the limited number of EBV(+) NSCLC cases analyzed), a definitive association between EBV and the subset of NSCLC cannot be confidently established at this moment. Further study with inclusion of more EBV(+) NSCLC patients will surely help us solve this puzzle.

## 4. Materials and Methods

### 4.1. Sequencing Data Set Acquisition

Controlled access RNA-seq data from 1017 non-small cell lung cancer samples and 110 paired adjacent normal lung tissue samples generated through the National Institutes of Health (NIH), The Cancer Genome Atlas (TCGA) project were obtained from the Cancer Genomics Hub and Genomic Data Commons (https://gdc.cancer.gov). Demographic and clinical data for each sample is available through the GDC data portal (https://portal.gdc.cancer.gov). Briefly, surgically removed samples were obtained from 9 countries (including the United States, Germany, Australia, Canada, Russia, Switzerland, Ukraine, Romania, and Vietnam) with no previous treatment. 

### 4.2. RNA CoMPASS Analysis

The RNA CoMPASS is an automated computational pipeline that seamlessly analyzes RNA-seq data sets [28,29,35]. Briefly, to reduce the run time and random-access memory requirements incurred during the running of the BLAST component of the pipeline, 20 million reads (around 1/3 of the total reads) were extracted from each sample using the Unix split command. The extracted reads were then deduplicated using an in-house deduplication algorithm to remove PCR duplication. The deduplicated reads were subsequently aligned to the human reference genome, hg19 (Genome Reference Consortium GRCH37), plus a splice junction database (which was generated using the make transcriptome application from the Useq [76]; splice junction radius set to the read length minus 4) using the Novoalign version 3.00.05 (Novocraft, Selangor, Malaysia) (-o SAM, default options) to identify human sequences. Unmapped sequencing reads were then isolated and subjected to consecutive BLAST (version 2.2.27 [77], default options) searches against the Human RefSeq RNA database and then to the NCBI NT database to pinpoint reads corresponding to known exogenous organisms [78]. Results from the NT BLAST searches were then filtered to eliminate matches with an E-value of greater than 1 × 10^−5^. The results were then processed by the taxonomic classifier software MEGAN 4 (version 4.70.4 [79]) for visualization of taxonomic classifications within the analyzed specimens. The RNA CoMPASS was run in parallel on three Intel Xeon Mac Pro workstations (with dual 12-core 2.66GHz CPUs and 64–96 GB of memory each).

### 4.3. Human and EBV Transcriptome Analysis

Raw sequencing reads were aligned to a reference genome containing a human genome (hg38; Genome Reference Consortium GRCH38) plus a modified Akata-EBV genome (Akata-NCBI accession number KC207813.1 [26]). The alignments were conducted using the Spliced Transcripts Alignment to a Reference (STAR) aligner version 2.5.3 (-clip5pNbases 6, default options) [80] and were subjected to visual inspection using the Integrative Genomics Viewer (IGV) genome browser [81]. Transcript data from STAR were then analyzed using the RSEM software (version 1.3.0 [82]) for quantification of human and EBV gene expression. Signal maps (i.e., the total number of reads covering each nucleotide position) were generated using the IGV tools, and read coverage maps were visualized using the IGV genome browser [81]. The EB-Seq software [40] was utilized to call statistically differentially-expressed genes using a false discovery rate (FDR) less than 0.05. 

### 4.4. Circular RNA (circRNA Backsplice Junction) Analysis

CircRNA candidates were identified by the back-splicing junctions. Briefly, raw sequence data were analyzed by the find_circ pipeline [83] using a reference genome containing a human genome (hg38; Genome Reference Consortium GRCH38) plus a modified Akata-EBV genome (Akata-NCBI accession number KC207813.1 [26]) with default parameters. 

### 4.5. Dimension Reduction, Correlation and Cluster Analysis of Human and EBV Expression Data

(i) Principal component analysis (PCA). PCA is a type of unsupervised dimension reduction method. It generates latent variables that are classified as principal components (PCs). The first principal component is a linear combination of the original variables that incorporates the greatest sources of variation within the datasets. The second and subsequent principal components are more latent variables which explain the greatest sources of variation that are left over beyond the first PC and lie orthogonal to it. To evaluate the variation between samples, we have utilized the PCA package (R version 3.4.1, the R Foundation, Vienna, Austria) (default settings), and analyzed both EBV and human gene expression data from 4 EBV(+) NSCLC datasets. The 2D plots were generated using the plot package (R version 3.4.1). (ii) Correlation analysis. The Pearson correlation coefficients were calculated by comparing both the human and EBV gene expression data of EBV(+) NSCLC samples using the correlation package (R version 3.4.1) with the default settings. Correlation plots were generated using the corrplot R package. (iii) Unsupervised hierarchical cluster analysis. The unsupervised hierarchical cluster analysis was performed using the pheatmap package with the default settings. The heatmaps and dendrograms were visualized using the pheatmap package with the default settings. 

### 4.6. Deconvolution of Immune Cell Infiltration in the Tumor Tissue

The CIBERSORT software [47] is a linear vector regression based machine learning approach and it is used to predict the proportions of immune cell subsets in tumor samples. Since the default CIBERSORT matrix panel is derived from the microarray data, it is thus not ideal to deconvole the RNA-seq data of tissue samples. To improve the accuracy of the deconvolution analyses, we utilized the CIBRESORT algorithm to build our custom CIBERSORT matrix panel of signature gene expression by using the gene expression data from the RNA-seq data sets of 18 immune cell subsets (NIH SRA# ERP004883, SRP075118, ERP013700, SRP059695, SRP066152, SRP066242). The gene expression data of EBV(+) LUSC samples were then used as input to infer proportions of 18 types of immune cells in the tumor tissue samples. 

### 4.7. Ingenuity Pathway Analysis (IPA)

Differentially expressed genes between the EBV-high and EBV-low lung squamous cell carcinoma samples (false discovery rate (FDR) < 0.05) were identified by the EB-Seq software and used as input for the IPA’s Core Analysis including both the downstream effects analyses and the upstream regulators analyses [84]. The downstream effects analyses were used to identify the biological processes and functions that are causally affected by the gene expression changes. The upstream regulator analyses were used to determine the molecules upstream of the genes that explain the altered gene expression. The Z-score is a value calculated by the Z-score algorithm of the IPA. The Z-score is utilized to predict the direction of change for a biological function or the activation state of the upstream regulator. The Z-score is calculated based on the uploaded gene expression pattern that is upstream to the biological function and downstream to an upstream regulator. A biological function is increased or an upstream regulator is activated if the Z-score is > 0. A biological function is decreased or an upstream regulator is inhibited if the Z-score is < 0. 

### 4.8. EBERs In Situ Hybridization

The formalin-fixed paraffin-embedded (FFPE) lung cancer tissue array was obtained (Biomax, Derwood, MD, USA, catalog no. BC041115d). EBERs (EBER1 and EBER2) ISH was performed using the HistoSonda EBER XISH Probes kit (American MasterTech, Lodi, CA, USA). Briefly, the FFPE tissue sections were deparaffinized, rehydrated in a graded solution of xylene and alcohol, and deproteinized with proteinase K. Samples were incubated with a digoxigenin EBER probe and washed with deionized water and 1× PBS. They were incubated first with anti-digoxin antibody and anti-mouse horse peroxidase antibody and subsequently with 3,3′-diaminobenzidine (Biocare Medical, Pacheco, CA, USA), counterstained with hematoxylin (Sigma, St. Louis, MO, USA), and washed again with 1× PBS. Slides were then dehydrated in a graded solution of xylene and alcohol and subsequently sealed with the VectaMount permanent mounting medium (Vector Laboratories, Burlingame, CA, USA). Slides were scanned with an Aperio CS2 digital pathology scanner, and images were obtained with Aperio ImageScope software (version 12.3.2.8013, Leica, Buffalo Grove, IL, USA) with 40× magnification.

### 4.9. Histopathology Images of TCGA Lung Cancer Samples

Hemotoxylin and eosin (H&E) stained histopathology images of the EBV(+) lung cancer samples of the TCGA cohort were obtained from the Genomics Data Commons (https://gdc.cancer.gov). All the tumor samples were collected by surgical excision. Representative images were generated using the Aperio ImageScope software (version 12.3.2.8013, Leica, Buffalo Grove, IL, USA) with 20× magnification.

### 4.10. Cell Culture

NCI-H1703 is a lung squamous cell carcinoma cell line and was purchased from the ATCC (Catalog number CRL-5889, Manassas, VA, USA). Cells were grown in RPMI 1640 medium (ThermoFisher Scientific, Waltham, MA, USA; catalog number SH30027) plus 10% fetal bovine serum (FBS; Invitrogen-Gibco, Carlsbad, CA, USA; catalog number 10437-028) with 0.5% pen-strep (Invitrogen-Gibco, Carlsbad, CA, USA; catalog number 15070-063) at 37 °C in a humidified 5% CO_2_ incubator. 

### 4.11. DNA Transfection

NCI-H1703 cells were seeded on either 6-well plates or chamber slides in RPMI 1640 medium supplemented with 10% FBS 1 day before transfection. On the day of transfection, DNA plasmids carrying either recombinant EBV M81 strain (rM81; a kind gift from Henri-Jacques Delecluse, [85]) or B95.8 strain (rB95.8; a kind gift from Wolfgang Hammerschmidt, [86]) or control pUHD10 plasmid were transfected into NCI-H1703 cells using the TransIT-X2 kit (Mirus, Madison, WI, USA; catalog number MIR6003) according to the vendor’s protocols. Cells were harvested 48 h later for the subsequent analyses.

### 4.12. RNA Extraction

Total cellular RNAs were isolated using the miRNeasy minikit (Qiagen, Germantown, MD, USA; catalog number 217004) according to the vendors’ protocols and treated with RNase-free DNase (Qiagen, Germantown, MD, USA; catalog number 79254) according to the vendor’s protocol. The quantity of the isolated RNA was further analyzed using a NanoDrop 2000 spectrophotometer (ThermoFisher Scientific, Waltham, MA, USA). RNA quality was examined by running RNA on a 1% agarose gel with ethidium bromide using a BioRad gel documentation system. 

### 4.13. Real-Time RT-PCR Analysis

Total RNA was reverse transcribed using the iScript cDNA synthesis kit for reverse transcription-PCR (RT-PCR) (BioRad, Hercules, CA, USA; catalog number 4106228). Random hexamers were used along with 1 µg of RNA in a 20-µL reaction volume according to the manufacturer’s instructions. For the incubation steps (25 °C for 5min followed by 46 °C for 20 min), a T100 thermal cycler (BioRad, Hercules, CA, USA) was used. The resulting cDNA was subjected to quantitative (real-time) PCR using sequence-specific forward and reverse primers (Integrated DNA Technologies, Coralville, Iowa, USA). For real-time PCR, 1 µL of the resulting cDNA was used in a 10-µL reaction volume that included 5 µL of Sybr green (BioRad, Hercules, CA, USA; catalog number 64213937) and a 500 nM concentration each of forward and reverse primers. Amplification was carried out using the following conditions: 95 °C for 3 min followed by 40 cycles of 95 °C for 15 s and 60 °C for 60 s. Melt curve analysis was performed at the end of every qRT-PCR run. Samples were tested in triplicates. No-template controls were included in each PCR run. PCRs were performed on a Bio-Rad CFX96 real-time system, and data analysis was performed using CFX Manager 3.0 software (BioRad, Hercules, CA, USA). Relative detection levels were calculated by normalizing with the glyceraldehyde-3-phosphate dehydrogenase (GAPDH) gene as a reference gene. Primer sequences for VISTA forward, ACCACCACTCGGAGCACAGG; reverse, TTGTAGACCAGGAGCAGGATGAGG; IDO forward, AGCCCTTCAAGTGTTTCACCAA; reverse, GCCTTTCCAGCCAGACAAATATA; BTLA forward, CATCTTAGCAGGAGATCCCTTTG; reverse, GACCCATTGTCATTAGGAAGCA; PD-1 forward, CGTGGCCTATCCACTCCTCA; reverse, ATCCCTTGTCCCAGCCACTC; CTLA-4 forward, AGCCAGGTGACTGAAGTCTG; reverse, CATAAATCTGGGTTCCGTTG; LAG3 forward, GCGGGGACTTCTCGCTATG; reverse, GGCTCTGAGAGATCCTGGGG; EBER forward, GGACCTACGCTGCCCTAG; reverse, CAGCTGGTACTTGACAGA; BZLF1 forward, CGACGTACAAGGAAACCACTAC; reverse, GAAGCCACCCGATTCTTGTAT; RPMS1 forward, CTAGTGCTGCATGGGCTCCTC; reverse, TGCAGATATCCTGCGTCCTCT; GAPDH forward, CCAAGGTCATCCATGACAACT; reverse, ATCACGCCACAGTTTCCC.

### 4.14. Fluorescence Microscopy Analysis

On the chamber slides, cells were fixed with 3.7% formaldehyde for 15 min at the room temperature. Fixed cells were then washed with 1× PBS for 5 min for 3 times. Nuclei were then counterstained for 15 min with NucBlue reagent (ThermoFisher scientific, Waltham, MA, USA; catalog number R37605) at the room temperature. Cells were then washed with 1× PBS for 5 min for 3 times. For fluorescence microscopy, slides were examined with a Nikon ECLIPSE 80i microscope (Nikon, Melville, NY, USA).

## 5. Conclusions

Overall, our current study strongly indicates that EBV is not a major carcinogen for LC in general, but EBV may play a critical role to promote the development of a subset of lung squamous cell carcinoma and lung adenocarcinoma cases. Our data also led to significant insights into the EBV-host interactions and the mechanisms through which EBV promotes lung carcinogenesis.

## Figures and Tables

**Table 1:** Listing of EBV backsplicing read counts in the EBV-high NSCLC sample.

Chromosome	Coord 1	Coord 2	Gene/Locus	Junction Counts
EBV Akata Strain	47242	47383	RPMS1	1
EBV Akata Strain	47516	47631	RPMS1	2
EBV Akata Strain	51610	51683	RPMS1	1

**Table 2:** Top 15 activated canonical pathways detected in the EBV-high sample by the Ingenuity pathway analysis (ranked by Z-score).

Canonical Pathways	Z-Score
Role of BRCA1 in DNA Damage Response	2.6
HIPPO signaling	2.3
TNFR1 Signaling	1.8
TNFR2 Signaling	1.7
CDK5 Signaling	1.5
Cell Cycle: G2/M DNA Damage Checkpoint Regulation	1.5
Sirtuin Signaling Pathway	1.5
Sonic Hedgehog Signaling	1.4
Glutamate Receptor Signaling	1.3
Death Receptor Signaling	1.0
Calcium Transport I	1.0
p53 Signaling	0.7
Fatty Acid alpha-oxidation	0.7
Th2 Pathway	0.7
Cell Cycle: G1/S Checkpoint Regulation	0.6

**Table 3:** Top 15 inhibited canonical pathways detected in the EBV-high sample by the Ingenuity pathway analysis (ranked by Z-score).

Canonical Pathways	Z-Score
Oxidative Phosphorylation	−3.3
Leukocyte Extravasation Signaling	−3.2
ILK Signaling	−3.2
TGF-beta Signaling	−3.1
IL-8 Signaling	−2.9
HMGB1 Signaling	−2.7
fMLP Signaling in Neutrophils	−2.5
Neuregulin Signaling	−2.5
BMP signaling pathway	−2.5
EIF2 Signaling	−2.4
NRF2-mediated Oxidative Stress Response	−2.4
B Cell Receptor Signaling	−2.2
Regulation of eIF4 and p70S6K Signaling	−2.1
CXCR4 Signaling	−2.1
Regulation of Cellular Mechanics by Calpain Protease	−2.1

**Table 4:** Top 15 activated upstream regulators detected in the EBV-high sample by the Ingenuity pathway analysis (ranked by Z-score).

Upstream Regulator	Z-Score
IFNL1	3.6
miR-21-5p (and other miRNAs w/seed AGCUUAU)	3.5
miR-155-5p (miRNAs w/seed UAAUGCU)	3.3
NANOG	2.9
PML	2.9
NEUROG1	2.8
HSF1	2.7
GSK3B	2.7
KDM5B	2.5
miR-17-5p (and other miRNAs w/seed AAAGUGC)	2.4
miR-30c-5p (and other miRNAs w/seed GUAAACA)	2.4
ZNF281	2.4
MSC	2.3
SIN3A	2.2
NR5A1	2.2

**Table 5:** Top 15 inhibited upstream regulators detected in the EBV-high sample by the Ingenuity pathway analysis (ranked by Z-score).

Upstream Regulator	Z-Score
TGFB3	−4.3
IL1A	−3.9
TGFB1	−3.8
CSF1	−3.6
IL6	−3.5
CSF2	−3.3
IGFBP2	−3.3
IL4	−3.1
SMAD3	−2.9
YAP1	−2.8
SMARCA4	−2.7
KLF6	−2.7
ELF4	−2.6
TGFB2	−2.6
ATF4	−2.6

**Table 6:** Clinical information of the EBV(+) NSCLC samples.

Patient ID	Diagnosis	Gender	Race	TNM Stage	Tumor Stage	Smoking History	Age	Source
Rln060065-C11	LUSC	FEMALE	N/A	T2-N2-M0	Stage IIIA	N/A	51	Biomax
Rln120096-D7	Adenosquamous	FEMALE	N/A	T2-N0-M0	Stage IB	N/A	64	Biomax
Rln060480-H6	LUAD	MALE	N/A	T2-N0-M0	Stage IB	N/A	49	Biomax
TCGA-96-A4JL	LUSC	FEMALE	ASIAN	T2a-N1-M0	Stage IIA	Lifelong Non-smoker	78	TCGA
TCGA-69-8255	LUAD	MALE	WHITE	T1a-N0-M0	Stage IA	smoker	71	TCGA
TCGA-98-7454	LUSC	MALE	WHITE	T2a-N0-M0	Stage IB	smoker	73	TCGA
TCGA-66-2769	LUSC	MALE	N/A	T4-N0-M0	Stage IIIB	smoker	75	TCGA

## Supplementary Materials

The following are available online at https://www.mdpi.com/2072-6694/11/6/759/s1, Figure S1: Unsupervised hierarchical cluster analysis of EBV(+) NSCLC and EBV(+) nasopharyngeal carcinoma (NPC) data sets. Cellular gene expression data of EBV(+) NSCLC and EBV(+) NPC samples were subjected to the unsupervised hierarchical cluster analysis using the pheatmap R package with the default settings. The dendrogram was then visualized using the pheatmap R package with the default settings, Figure S2: The EBV transcriptome in lung cancer. EBV genome coverage data for the four EBV(+) NSCLC. Data was displayed using the Integrative Genomics Viewer (IGV) using the modified Akata-EBV genome. The modified EBV Akata genome was split between the BBLF2/3 and the BGLF3.5 lytic genes rather than at the terminal repeats to accommodate coverage of splice junctions for the latency membrane protein LMP2. The *y* axis represents the number of reads at each nucleotide position. Blue features represent lytic genes, red features represent latent genes, green features represent potential noncoding genes, aquamarine features represent microRNAs, and black features represent non-gene features (e.g., repeat regions), Figure S3: Alternative splicing in the EBV LMP2A in EBV-high NSCLC. RNA-seq data of the EBV-high NSCLC were analyzed using the STAR aligner and were aligned to the modified Akata-EBV genome to obtain splice junction information. Junctions were visualized using the Integrative Genomic Viewer (IGV). The thickness of the red junction features correlates with the number of reads for the respective junction. The number of junction spanning reads for each junction is indicated above each junction feature. Inset: Detailed read coverage data for the 5′ flanking region of the second exon of EBV LMP2A, Figure S4: Representative images of hematoxylin and eosin staining of EBV(+) NSCLC and adjacent normal lung samples. Arrowheads point to the infiltrating immune cells. Scale bar: 50 µm, Figure S5: EBV induces cellular checkpoint molecules in lung squamous cell carcinoma cells. (A) NCI-H1703 cells were transfected with DNA plasmids carrying either recombinant EBV M81 strain (rM81) or B95.8 strain (rB95.8) or the control pUHD10 (CNTL) plasmids. Forty-eight hours post-transfection, cells were examined by fluorescence microscopy. Both the recombinant EBV strains rM81 and rB95.8 carry the GFP gene (Green Fluorescence Protein) which can be constitutively expressed. Nuclei were visualized by NucBlue (Hoeschst) staining. (B and C) Total RNA was extracted from the transfected NCI-H1703 cells and subjected to the qRT-PCR analysis. GAPDH was analyzed as a reference. The expression of cellular checkpoint molecules and EBV genes was determined by the comparative *CT* method (2^*−ΔΔCT*^), Table S1: EBV read counts in EBV(+) non-small cell lung cancer (NSCLC), Table S2: Type I EBV was detected in EBV(+) NSCLC. Data sets of EBV(+) JY and EBV(+) P3HR1 cells which carry either Type I or Type II EBV strain were analyzed and used as positive controls, Table S3: List of 184 analyzed human lung cancer cell lines from the Cancer Cell Line Encyclopedia (CCLE) cohort.
