# Supplementary material for: Detection of Epstein-Barr Virus Infection in Non-Small Cell Lung Cancer
Source: Cancers (Basel). 2019 May 31;11(6):759. doi: 10.3390/cancers11060759 (PMC6627930; doi:10.3390/cancers11060759)
Supplement: Supplementary file 1 [file cancers-11-00759-s001.pdf]

FIGURE LEGENDS – SUPPLEMENTARY MATERIAL

Table S1. EBV read counts in EBV(+) non-small cell lung cancer (NSCLC).

Table S2. Type I EBV was detected in EBV(+) NSCLC. Data sets of EBV(+) JY and EBV(+) P3HR1 cells which carry either Type I or Type II EBV strain were analyzed and used as positive controls.

Figure S1. Unsupervised hierarchical cluster analysis of EBV(+) NSCLC and EBV(+) nasopharyngeal carcinoma (NPC) data sets. Cellular gene expression data of EBV(+) NSCLC and EBV(+) NPC samples were subjected to the unsupervised hierarchical cluster analysis using the pheatmap R package with the default settings. The dendrogram was then visualized using the pheatmap R package with the default settings.

Figure S2. The EBV transcriptome in lung cancer. EBV genome coverage data for the four EBV(+) NSCLC. Data was displayed using the Integrative Genomics Viewer (IGV) using the modified Akata-EBV genome. The modified EBV Akata genome was split between the BBLF2/3 and the BGLF3.5 lytic genes rather than at the terminal repeats to accommodate coverage of splice junctions for the latency membrane protein LMP2. The *y* axis represents the number of reads at each nucleotide position. Blue features represent lytic genes, red features represent latent genes, green features represent potential noncoding

genes, aquamarine features represent microRNAs, and black features represent non-gene features (e.g., repeat regions).

Figure S3. Alternative splicing in the EBV LMP2A in EBV-high NSCLC. RNA-seq data of the EBV-high NSCLC were analyzed using the STAR aligner and were aligned to the modified Akata-EBV genome to obtain splice junction information. Junctions were visualized using the Integrative Genomic Viewer (IGV). The thickness of the red junction features correlates with the number of reads for the respective junction. The number of junction spanning reads for each junction is indicated above each junction feature. Inset: Detailed read coverage data for the 5' flanking region of the second exon of EBV LMP2A.

Figure S4. Representative images of hematoxylin and eosin staining of EBV(+) NSCLC and adjacent normal lung samples. Arrowheads point to the infiltrating immune cells. Scale bar: 50 μm.

Figure S5. EBV induces cellular checkpoint molecules in lung squamous cell carcinoma cells. (A) NCI-H1703 cells were transfected with DNA plasmids carrying either recombinant EBV M81 strain (rM81) or B95.8 strain (rB95.8) or the control pUHD10 (CNTL) plasmids. Forty-eight hours post-transfection, cells were examined by fluorescence microscopy. Both the recombinant EBV strains rM81 and rB95.8 carry the GFP gene (Green Fluorescence Protein) which can be constitutively expressed. Nuclei were visualized by NucBlue (Hoeschst)

staining. (B and C) Total RNA was extracted from the transfected NCI-H1703 cells and subjected to the qRT-PCR analysis. GAPDH was analyzed as a reference. The expression of cellular checkpoint molecules and EBV genes was determined by the comparative C_T method ($2^{-\Delta\Delta CT}$).

Table S3. List of 184 analyzed human lung cancer cell lines from the Cancer Cell Line Encyclopedia (CCLE) cohort.

Patient_ID	Total number of EBV read	Total number of human read	EBV reads per million mapped human reads
TCGA-96-A4JL	24874	59652672	416.98
TCGA-69-8255	94	117763296	0.80
TCGA-98-7454	57	79659303	0.72
TCGA-66-2769	30	71251428	0.42

Table S1. EBV read counts in EBV(+) non-small cell lung cancer (NSCLC).

Sample	Type I EBNA2/3	Type II EBNA2/3	EBV Strain
TCGA-96-A4JL	+	-	Type I
JY	+	-	Type I
P3HR1	+	-	Type II

Table S2. Type I EBV was detected in EBV(+) NSCLC. Data sets of EBV(+) JY and EBV(+) P3HR1 cells which carry either Type I or Type II EBV strain were analyzed and used as positive controls.

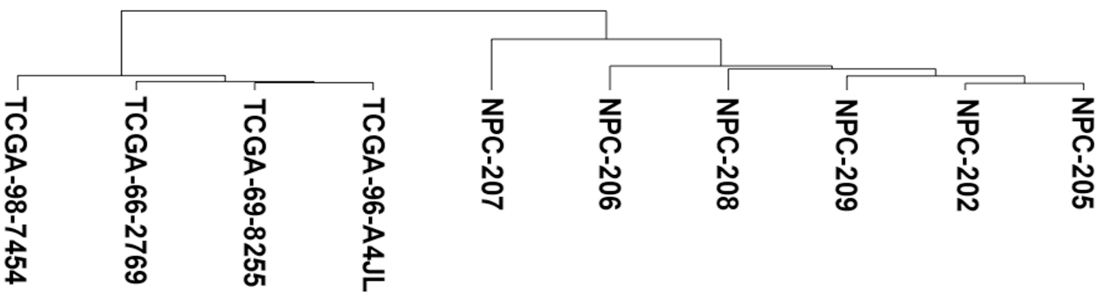

Figure S1. Unsupervised hierarchical cluster analysis of EBV(+) NSCLC and EBV(+) nasopharyngeal carcinoma (NPC) data sets. Cellular gene expression data of EBV(+) NSCLC and EBV(+) NPC samples were subjected to the unsupervised hierarchical cluster analysis using the pheatmap R package with the default settings. The dendrogram was then visualized using the pheatmap R package with the default settings.

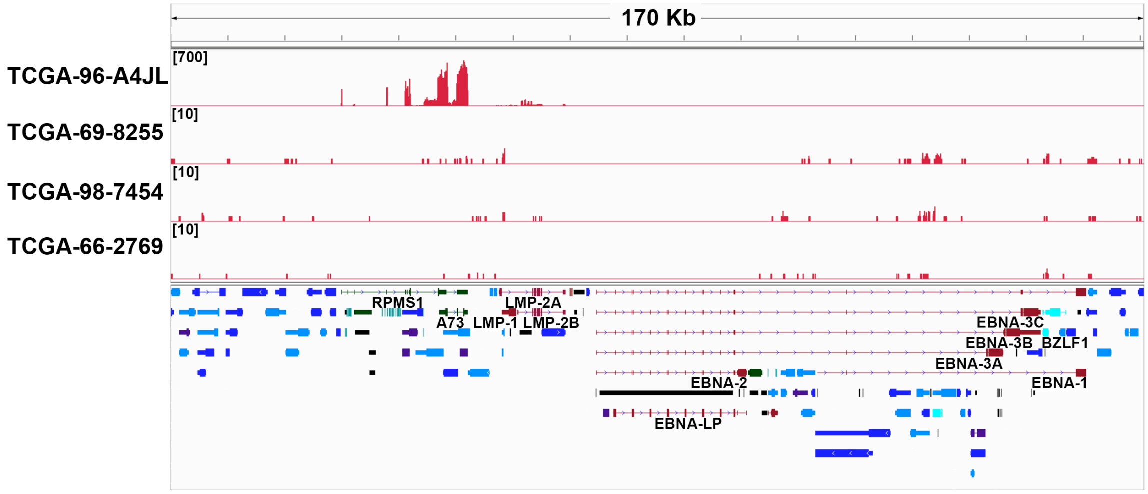

Figure S2. The EBV transcriptome in lung cancer. EBV genome coverage data for the four EBV(+) NSCLC. Data was displayed using the Integrative Genomics Viewer (IGV) using the modified Akata-EBV genome. The modified EBV Akata genome was split between the BBLF2/3 and the BGLF3.5 lytic genes rather than at the terminal repeats to accommodate coverage of splice junctions for the latency membrane protein LMP2. The *y* axis represents the number of reads at each nucleotide position. Blue features represent lytic genes, red features represent latent genes, green features represent potential noncoding genes, aquamarine features represent microRNAs, and black features represent non-gene features (e.g., repeat regions).

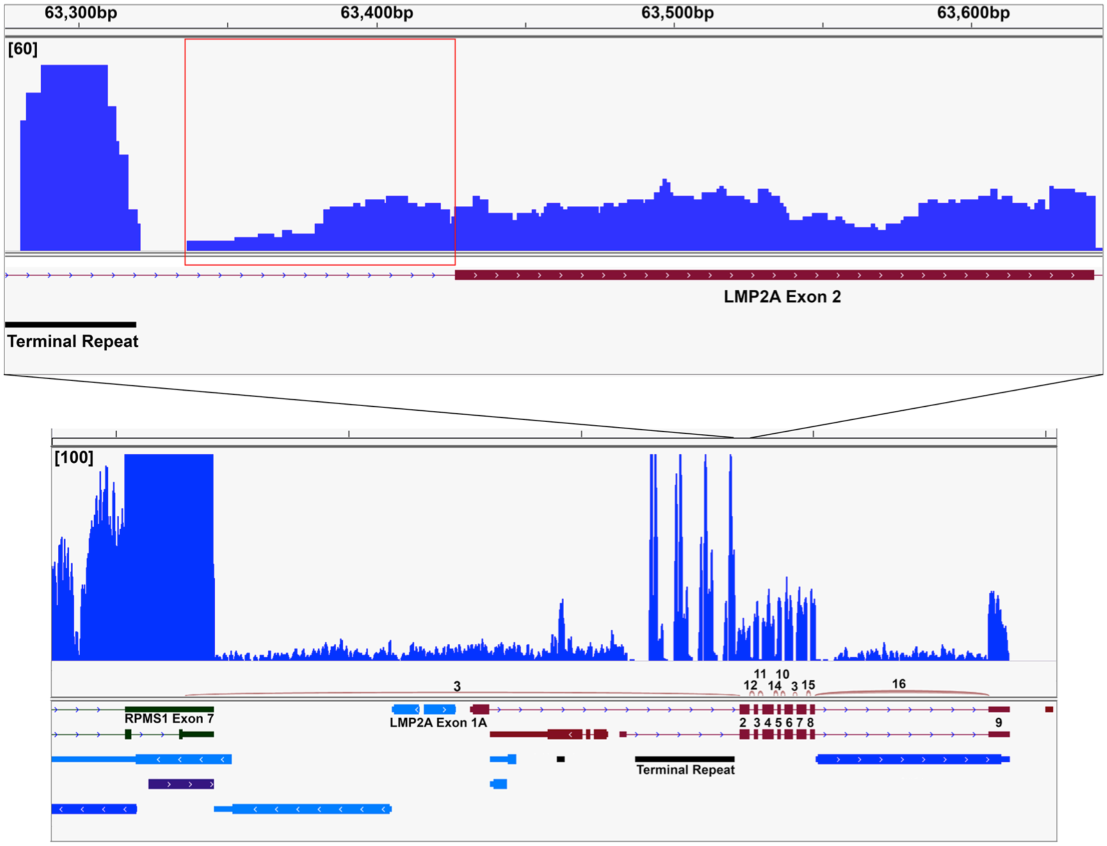

Figure S3. Alternative splicing in the EBV LMP2A in EBV-high NSCLC. RNA-seq data of the EBV-high NSCLC were analyzed using the STAR aligner and were aligned to the modified Akata-EBV genome to obtain splice junction information. Junctions were visualized using the Integrative Genomic Viewer (IGV). The thickness of the red junction features correlates with the number of reads for the respective junction. The number of junction spanning reads for each junction is indicated above each junction feature. Inset: Detailed read coverage data for the 5' flanking region of the second exon of EBV LMP2A.

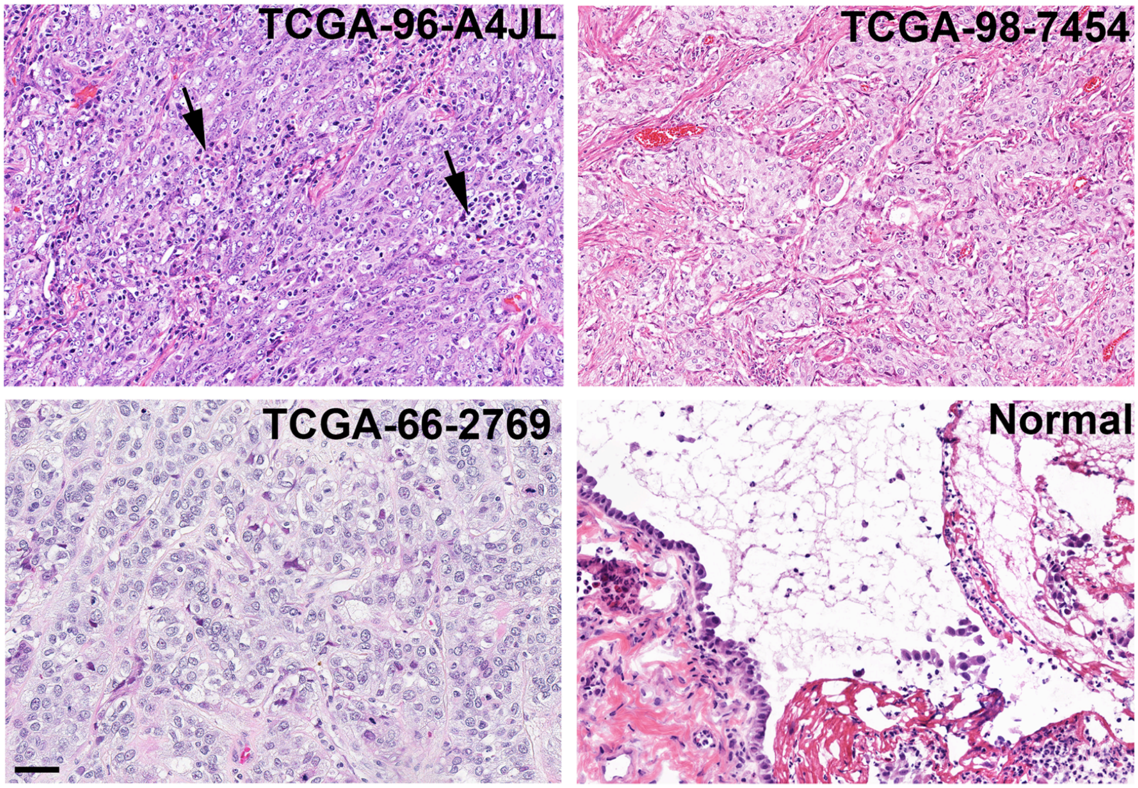

Figure S4. Representative images of hematoxylin and eosin staining of EBV(+) NSCLC and adjacent normal lung samples. Arrowheads point to the infiltrating immune cells. Scale bar: 50 μm.

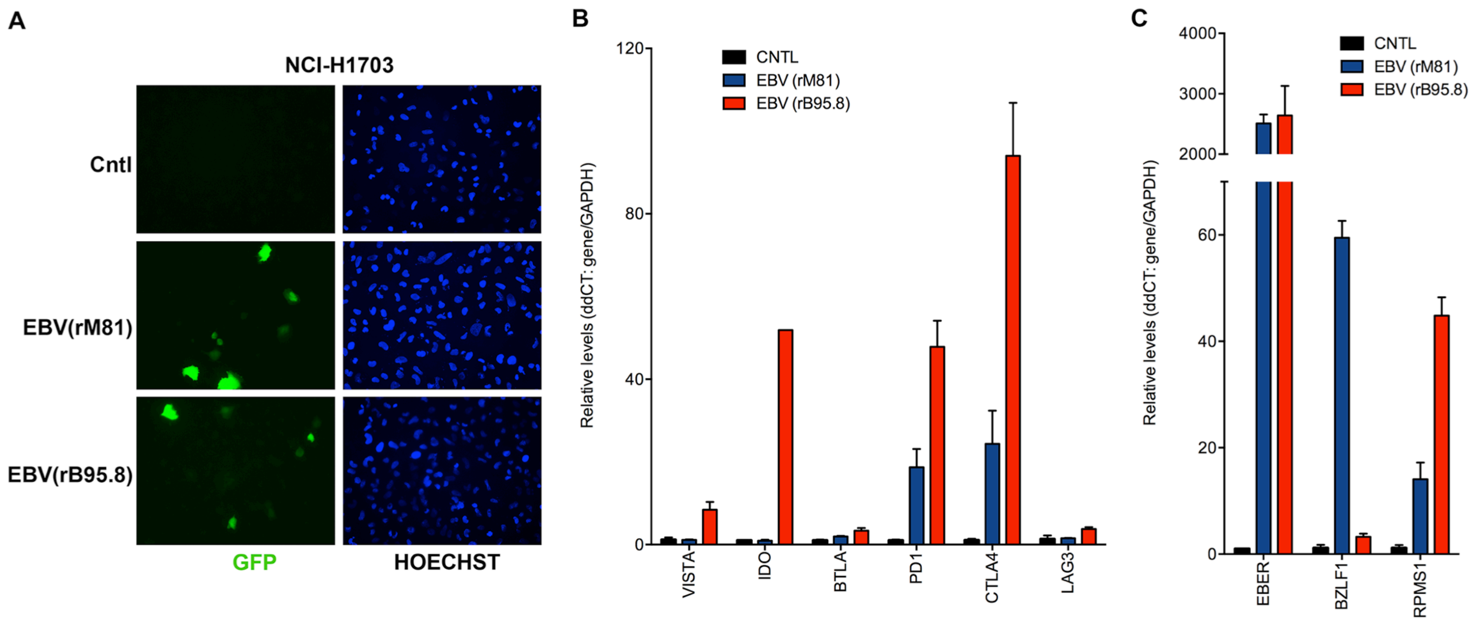

Figure S5. EBV induces cellular checkpoint molecules in lung squamous cell carcinoma cells. (A) NCI-H1703 cells were transfected with DNA plasmids carrying either recombinant EBV M81 strain (rM81) or B95.8 strain (rB95.8) or the control pUHD10 (CNTL) plasmids. Forty-eight hours post-transfection, cells were examined by fluorescence microscopy. Both the recombinant EBV strains rM81 and rB95.8 carry the GFP gene (Green Fluorescence Protein) which can be constitutively expressed. Nuclei were visualized by NucBlue (Hoeschst) staining. (B and C) Total RNA was extracted from the transfected NCI-H1703 cells and subjected to the qRT-PCR analysis. GAPDH was analyzed as a reference. The expression of cellular checkpoint molecules and EBV genes was determined by the comparative C_T method ($2^{-\Delta\Delta CT}$).

Table S3. List of 184 analyzed human lung cancer cell lines from the Cancer Cell Line Encyclopedia (CCLE) cohort.

Cell_Line_ID	Gender	Primary Site	Histology Subtype
A549	Male	lung	adenocarcinoma
ABC-1	Male	lung	non_small_cell_carcinoma
BEN	Male	lung	non_small_cell_carcinoma
CAL-12T	Male	lung	non_small_cell_carcinoma
Calu-1	Male	lung	squamous_cell_carcinoma
Calu-3	Male	lung	adenocarcinoma
Calu-6	Female	lung	undifferentiated_carcinoma
ChaGo-K-1	Male	lung	bronchogenic_carcinoma
COLO_668	Female	lung	small_cell_carcinoma
COR-L105	Male	lung	adenocarcinoma
COR-L23	Male	lung	large_cell_carcinoma
COR-L24	Male	lung	small_cell_carcinoma
COR-L279	Male	lung	small_cell_carcinoma
COR-L311	Male	lung	small_cell_carcinoma
COR-L47	Male	lung	small_cell_carcinoma
COR-L88	Male	lung	small_cell_carcinoma
COR-L95	Male	lung	small_cell_carcinoma
DM-3	Male	lung	Pleural_sarcomatoid_mesothelioma
DMS_114	Male	lung	small_cell_carcinoma
DMS_153	Male	lung	small_cell_carcinoma
DMS_273	Female	lung	small_cell_carcinoma
DMS_454	Male	lung	small_cell_carcinoma
DMS_53	Male	lung	small_cell_carcinoma
DMS_79	Male	lung	small_cell_carcinoma
DV-90	Male	lung	adenocarcinoma
EBC-1	Male	lung	squamous_cell_carcinoma
EKVX	Unknown	lung	adenocarcinoma
EPLC-272H	Male	lung	squamous_cell_carcinoma
HARA	Male	lung	squamous_cell_carcinoma
HCC-1171	Male	lung	non_small_cell_carcinoma
HCC-1195	Male	lung	mixed_adenosquamous_carcinoma
HCC-1359	Female	lung	large_cell_carcinoma
HCC-1438	Male	lung	large_cell_carcinoma
HCC-15	Male	lung	squamous_cell_carcinoma
HCC-1588	Female	lung	squamous_cell_carcinoma
HCC-1833	Unknown	lung	adenocarcinoma
HCC-2108	Male	lung	adenocarcinoma
HCC-2279	Female	lung	adenocarcinoma

HCC-33	Male	lung	small_cell_carcinoma
HCC-366	Female	lung	mixed_adenosquamous_carcinoma
HCC-44	Female	lung	adenocarcinoma
HCC-78	Male	lung	adenocarcinoma
HCC-95	Male	lung	squamous_cell_carcinoma
HCC2935	Male	lung	non_small_cell_carcinoma
HCC4006	Male	lung	adenocarcinoma
HCC827	Female	lung	adenocarcinoma
HLF-a	Female	lung	squamous_cell_carcinoma
HOP-62	Female	lung	adenocarcinoma
HOP-92	Male	lung	NSCLC
Hs_229.T	Male	lung	adenocarcinoma
Hs_618.T	Female	lung	adenocarcinoma
IA-LM	Male	lung	large_cell_carcinoma
IST-MES1	Female	lung	Pleural_epithelioid_mesothelioma
IST-MES2	Male	lung	Pleural_epithelioid_mesothelioma
JL-1	Male	lung	Pleural_epithelioid_mesothelioma
KNS-62	Male	lung	squamous_cell_carcinoma
LC-1F	Male	lung	squamous_cell_carcinoma
LCLC-103H	Male	lung	large_cell_carcinoma
LCLC-97TM1	Male	lung	large_cell_carcinoma
LK-2	Male	lung	squamous_cell_carcinoma
LOU-NH91	Female	lung	squamous_cell_carcinoma
LU65	Male	lung	non_small_cell_carcinoma
LU99	Male	lung	large_cell_carcinoma
LUDLU-1	Male	lung	squamous_cell_carcinoma
LXF-289	Male	lung	adenocarcinoma
MOR_CPR	Unknown	lung	adenocarcinoma
MPP_89	Male	lung	Pleural_epithelioid_mesothelioma
MSTO-211H	Male	lung	Pleural_biphasic_mesothelioma
NCI-H1048	Female	lung	small_cell_carcinoma
NCI-H1092	Male	lung	small_cell_carcinoma
NCI-H1105	Male	lung	small_cell_carcinoma
NCI-H1155	Male	lung	large_cell_carcinoma
NCI-H1184	Male	lung	small_cell_carcinoma
NCI-H1299	Male	lung	non_small_cell_carcinoma
NCI-H1339	Female	lung	small_cell_carcinoma
NCI-H1341	Female	lung	small_cell_carcinoma
NCI-H1355	Male	lung	adenocarcinoma
NCI-H1385	Female	lung	squamous_cell_carcinoma
NCI-H1395	Female	lung	adenocarcinoma

NCI-H1435	Female	lung	non_small_cell_carcinoma
NCI-H1436	Male	lung	small_cell_carcinoma
NCI-H1437	Male	lung	adenocarcinoma
NCI-H146	Male	lung	small_cell_carcinoma
NCI-H1563	Male	lung	adenocarcinoma
NCI-H1568	Female	lung	non_small_cell_carcinoma
NCI-H1573	Female	lung	adenocarcinoma
NCI-H1581	Male	lung	large_cell_carcinoma
NCI-H1618	Female	lung	small_cell_carcinoma
NCI-H1623	Male	lung	adenocarcinoma
NCI-H1648	Male	lung	adenocarcinoma
NCI-H1650	Male	lung	bronchioloalveolar_adenocarcinoma
NCI-H1651	Male	lung	adenocarcinoma
NCI-H1666	Female	lung	bronchioloalveolar_adenocarcinoma
NCI-H1693	Female	lung	adenocarcinoma
NCI-H1694	Male	lung	small_cell_carcinoma
NCI-H1703	Male	lung	squamous_cell_carcinoma
NCI-H1734	Female	lung	adenocarcinoma
NCI-H1755	Female	lung	adenocarcinoma
NCI-H1781	Female	lung	bronchioloalveolar_adenocarcinoma
NCI-H1792	Male	lung	adenocarcinoma
NCI-H1793	Female	lung	non_small_cell_carcinoma
NCI-H1838	Female	lung	non_small_cell_carcinoma
NCI-H1869	Male	lung	squamous_cell_carcinoma
NCI-H1876	Male	lung	small_cell_carcinoma
NCI-H1915	Female	lung	large_cell_carcinoma
NCI-H1930	Male	lung	small_cell_carcinoma
NCI-H1944	Female	lung	non_small_cell_carcinoma
NCI-H196	Male	lung	small_cell_carcinoma
NCI-H1963	Male	lung	small_cell_carcinoma
NCI-H1975	Female	lung	non_small_cell_carcinoma
NCI-H2009	Female	lung	adenocarcinoma
NCI-H2023	Male	lung	adenocarcinoma
NCI-H2029	Female	lung	small_cell_carcinoma
NCI-H2030	Male	lung	non_small_cell_carcinoma
NCI-H2052	Male	lung	Pleural_sarcomatoid_mesothelioma
NCI-H2066	Female	lung	small_cell_carcinoma
NCI-H2073	Female	lung	adenocarcinoma
NCI-H2081	Female	lung	small_cell_carcinoma
NCI-H2085	Male	lung	adenocarcinoma
NCI-H2087	Male	lung	adenocarcinoma

NCI-H209	Male	lung	small_cell_carcinoma
NCI-H2106	Male	lung	non_small_cell_carcinoma
NCI-H211	Female	lung	small_cell_carcinoma
NCI-H2110	Unknown	lung	non_small_cell_carcinoma
NCI-H2122	Female	lung	adenocarcinoma
NCI-H2126	Male	lung	adenocarcinoma
NCI-H2170	Male	lung	squamous_cell_carcinoma
NCI-H2171	Male	lung	small_cell_carcinoma
NCI-H2172	Female	lung	non_small_cell_carcinoma
NCI-H2196	Male	lung	small_cell_carcinoma
NCI-H2227	Male	lung	small_cell_carcinoma
NCI-H2228	Female	lung	adenocarcinoma
NCI-H226	Male	lung	squamous_cell_carcinoma
NCI-H2286	Female	lung	small_cell_carcinoma
NCI-H2291	Male	lung	adenocarcinoma
NCI-H23	Male	lung	non_small_cell_carcinoma
NCI-H2342	Male	lung	adenocarcinoma
NCI-H2347	Female	lung	adenocarcinoma
NCI-H2405	Male	lung	adenocarcinoma
NCI-H2444	Male	lung	non_small_cell_carcinoma
NCI-H2452	Male	lung	Pleural_biphasic_mesothelioma
NCI-H28	Male	lung	Pleural_sarcomatoid_mesothelioma
NCI-H292	Female	lung	mucoepidermoid_carcinoma
NCI-H322	Male	lung	adenocarcinoma
NCI-H3255	Female	lung	adenocarcinoma
NCI-H358	Male	lung	bronchioloalveolar_adenocarcinoma
NCI-H441	Male	lung	adenocarcinoma
NCI-H446	Male	lung	small_cell_carcinoma
NCI-H460	Male	lung	large_cell_carcinoma
NCI-H510	Male	lung	small_cell_carcinoma
NCI-H520	Male	lung	squamous_cell_carcinoma
NCI-H522	Male	lung	non_small_cell_carcinoma
NCI-H524	Male	lung	small_cell_carcinoma
NCI-H526	Male	lung	small_cell_carcinoma
NCI-H596	Male	lung	mixed_adenosquamous_carcinoma
NCI-H647	Male	lung	mixed_adenosquamous_carcinoma
NCI-H650	Male	lung	bronchioloalveolar_adenocarcinoma
NCI-H661	Male	lung	large_cell_carcinoma
NCI-H69	Male	lung	small_cell_carcinoma
NCI-H727	Female	lung	non_small_cell_carcinoma
NCI-H810	Male	lung	large_cell_carcinoma

NCI-H82	Male	lung	small_cell_carcinoma
NCI-H838	Male	lung	non_small_cell_carcinoma
NCI-H841	Male	lung	small_cell_carcinoma
NCI-H854	Unknown	lung	adenocarcinoma
NCI-H889	Female	lung	small_cell_carcinoma
PC-14	Unknown	lung	non_small_cell_carcinoma
RERF-LC-Ad1	Male	lung	adenocarcinoma
RERF-LC-Ad2	Male	lung	adenocarcinoma
RERF-LC-AI	Male	lung	squamous_cell_carcinoma
RERF-LC-KJ	Male	lung	non_small_cell_carcinoma
RERF-LC-MS	Unknown	lung	non_small_cell_carcinoma
RERF-LC-Sq1	Female	lung	squamous_cell_carcinoma
RS-5	Male	lung	Pleural_sarcomatoid_mesothelioma
SBC-5	Unknown	lung	small_cell_carcinoma
SCLC-21H	Male	lung	small_cell_carcinoma
SHP-77	Male	lung	small_cell_carcinoma
SK-LU-1	Female	lung	adenocarcinoma
SK-MES-1	Male	lung	squamous_cell_carcinoma
Sq-1	Unknown	lung	squamous_cell_carcinoma
SW_1271	Male	lung	small_cell_carcinoma
SW_1573	Female	lung	adnocarcinoma
SW_900	Male	lung	squamous_cell_carcinoma
T3M-10	Male	lung	large_cell_carcinoma
